# Research on Modeling of the Agile Satellite Using a Single Gimbal Magnetically Suspended CMG and the Disturbance Feedforward Compensation for Rotors

**DOI:** 10.3390/s121216964

**Published:** 2012-12-12

**Authors:** Peiling Cui, Ning Yan

**Affiliations:** 1 School of Instrumentation Science and Optoelectronics Engineering, Beihang University, Beijing 100191, China; E-Mail: n_yann@sina.com; 2 Science and Technology on Inertial Laboratory, Beijing 100191, China; 3 Research and Design Centre, China Academy of Launch Vehicle Technology, Beijing 100076, China

**Keywords:** Control Moment Gyroscope, dynamic modeling, feedforward compensation, magnetic bearing

## Abstract

The magnetically suspended Control Moment Gyroscope (CMG) has the advantages of long-life, micro-vibration and being non-lubricating, and is the ideal actuator for agile maneuver satellite attitude control. However, the stability of the rotor in magnetic bearing and the precision of the output torque of a magnetically suspended CMG are affected by the rapid maneuvers of satellites. In this paper, a dynamic model of the agile satellite including a magnetically suspended single gimbal control moment gyroscope is built and the equivalent disturbance torque effected on the rotor is obtained. The feedforward compensation control method is used to depress the disturbance on the rotor. Simulation results are given to show that the rotor displacement is obviously reduced.

## 1. Introduction

With the development of high resolution imaging on Earth, the requirement of satellite agility is increasing. The magnetically suspended Single Gimbal Control Moment Gyroscope (SGCMG) is chosen as the ideal actuator for its large output torque to perform rapid maneuver tasks by using the magnetic bearing instead of traditional mechanical bearing [1–3]. It has the advantage of high precision, long-life, micro-vibration and being non-lubricating [4]. It can be applied for agile maneuver satellite attitude control. For agile satellites, the slew rate is often at the level of 1∼10°/s [5], but when the satellite slews fast or the gimbal rotates rapidly, the rotor displacement will be increased and the precision of output torque will be decreased, even leading to rotor instability, so research on agile satellite attitude control using magnetically suspended SGCMGs is very important.

For the control of magnetic bearing systems, scholars have reported some research results [4–7]. For example, cross feedback control of magnetic bearing systems [6], filtered-X least mean square (FXLMS) algorithm for moving-gimbal effects [4], and the compound control for moving-gimbal effect compensation to increase the response of the gimbal system [7]. The research is all based on a static base, and the influence of rapid satellite maneuvers on the rotor displacement is not analyzed.

For the satellite attitude control based on a Control Moment Gyroscope (CMG), there are some research results [8–14]. Reference [12] gives a model of the CMG mechanism and the CMG output torque in body frame. In Reference [13], aiming at solving the problem of high precision and high stability attitude control of satellites, the influence of magnetically suspended rotor dynamic imbalance and static imbalance on the vibration of the base, and the stability of a magnetically suspended control system are investigated. The disturbance torque on the base resulting from the imbalance vibration is reduced. In Reference [14], a dynamic model of a spacecraft with a magnetically suspended single gimbal control moment gyroscope is given, but the slew rate of the satellite is limited at 10^−2^rad/s level (approximate 0.5°/s). How to use magnetically suspended SGCMGs for the agile maneuver attitude control of the satellite is not mentioned.

In this paper, the dynamic model of the agile satellite including a magnetically suspended single gimbal control moment gyroscope is built and the equivalent disturbance torque effected on the rotor is obtained. The feedforward compensation control method is used to depress the disturbance on the rotor. Simulation results are given to show that the rotor displacement is obviously decreased.

## 2. The Satellite Attitude Dynamic Model Including a Magnetically Suspended SGCMGs

**Figure 1.**
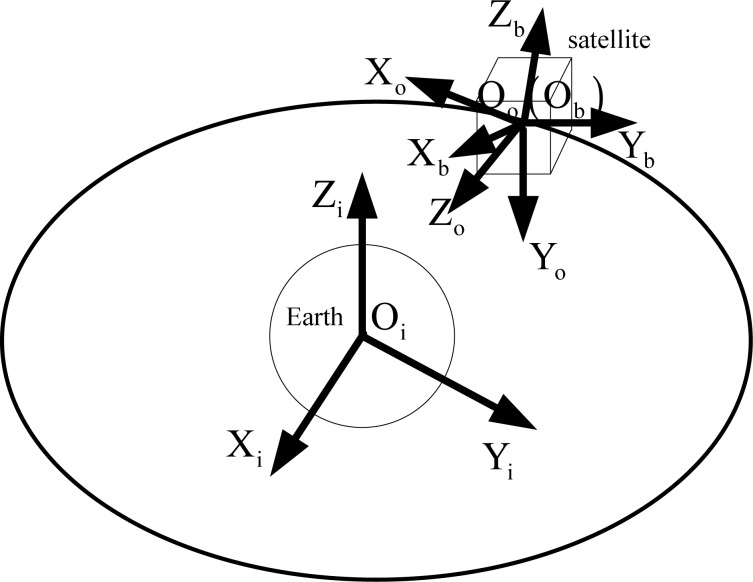
The relationship between the coordinate frames.

Different from the mechanical CMG, a magnetically suspended rotor is used in a magnetically suspended CMG. The translational position of the rotor relative to the magnetic bearing can be controlled actively. Besides the high-speed rotation degree, the magnetically suspended CMG has five more degrees of freedom than the mechanical CMG, and thus the modeling process is complex. Firstly, the coordinate frames are needed to be defined. In the following, *n* = 1, 2, 3, 4 denotes the *n*-th CMG. Figure 1 gives the relationship between inertial frame system, orbit frame, and satellite body frame.

F_i_: Inertia frame O_i_X_i_Y_i_ Z_i_. The origin is the Earth’s core, o_i_x_i_ points to the vernal equinox, while o_i_z_i_ points to the North Pole.
F_o_: Orbit frame O_o_X_o_Y_o_Z_o_. The origin is the centre of mass of the satellite, o_o_x_o_ and o_o_y_o_ represent the roll axis and the pitch axis, respectively, both locating in the orbital plane.
F_b_: Satellite body frame O_b_X_b_Y_b_Z_b_. The origin is the centre of mass of satellite. o_b_x_b_, o_b_y_b_ and o_b_z_b_ are along the principal axes of inertia of the satellite.
F_cmg,n_: The *n* -th CMG frame O_cmg,n_X_cmg,n_Y_cmg,n_Z_cmg,n_. Fixed with the satellite, but it is determined by configuration. O_cmg,n_ is the rotor centre of mass of the *n* -th magnetically suspended SGCMG, X_cmg,n_ is the gimbal axis, Y_cmg,n_ is the spin direction of the rotor when the gimbal angle is in zero position.
F_g,n_: The *n* -th gimbal frame O_g,n_X_g,n_Y_g,n_Z_g,n_. Fixed with the gimbal, the frame can rotate with the gimbal. When the gimbal angle is in zero position, the gimbal frame coincides with the CMG frame.
F_f ,n_: The *n* -th magnetic bearing installed frame O_f ,n_X_f ,n_Y_f ,n_Z_f ,n_. It is fixed with the gimbal frame. This frame is obtained by 45° rotation of the gimbal frame and about the Y_g,n_ axis.
F_r,n_ : The *n* -th rotor frame O_r,n_X_r,n_Y_r,n_Z_r,n_. It is fixed with the rotor. This frame does not spin with the rotor. O_f ,n_ coincides with O_r,n_ when the rotor is not suspended.
$C_{{\text{F}}_{1}}^{{\text{F}}_{2}}$: denotes the coordinate transformation matrix from frame F_1_ to frame F_2_.

In Section 2.1, the rotor dynamic model of single gimbal magnetically suspended CMG is analyzed. For convenience, the index *n* is omitted. For example, F_g_ represents the gimbal frame o_g_x_g_y_g_z_g_.

### 2.1. The Dynamic Model of Magnetically Suspended Rotor

The Euler equation is used to build the magnetically suspended rotor dynamic model. A magnetically suspended SGCMG consists of the rotor and the gimbal. The magnetic force is produced from the unique relation between the rotor and the bearing. In order to perform the satellite attitude control, the direction of angular momentum is changed through magnetic bearing torque by the gimbal rotation, and then the gyroscope torque is generated, and is transmitted to the satellite by the magnetic bearings.

**Figure 2.**
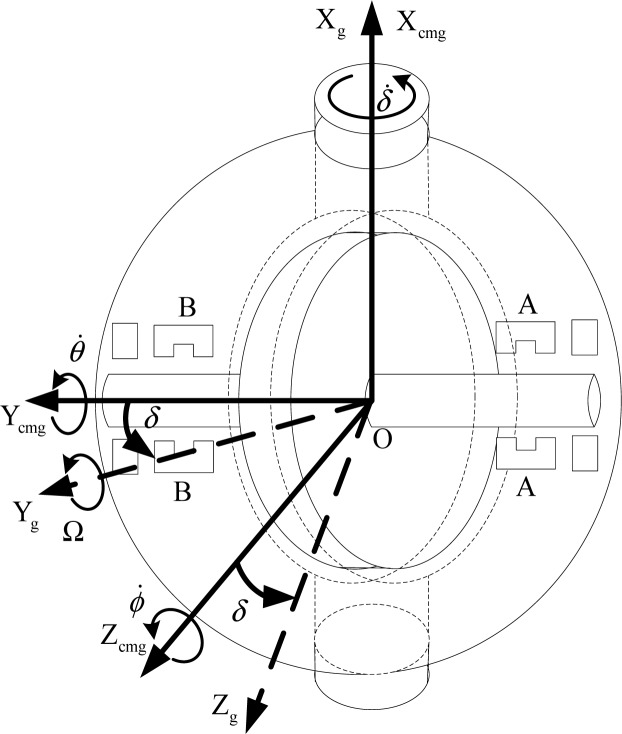
A sketch of the magnetically suspended SGCMG.

In this paper, the rotor is supposed to be asymmetric, and the dynamic and static vibration induced by the geometry shape are ignored. Only the force induced from the gimbal rotation of the CMG frame relative to the inertial frame is considered. The rotation relationship between CMG frame and the gimbal frame is given in Figure 2.

**Figure 3.**
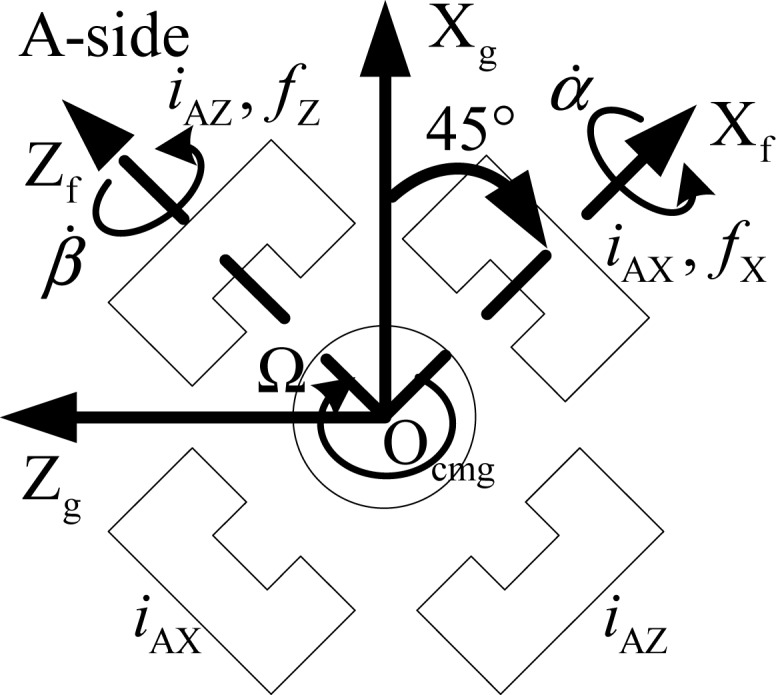
Sketch map of the magnetic bearing installation.

As shown in Figure 2, *δ̇*, *θ̇* and *ϕ̇* are the angular velocity of CMG frame relative to gimbal frame. *δ* is the rotation angle from CMG frame to gimbal frame. *δ̇* is the gimbal rate about X*_cmg_* axis. Figure 3 denotes the view from A-side about the Y*_cmg_*-direction in Figure 2. It shows the relationship between the gimbal frame and the installed magnetic bearing frame. *f*_X_ and *f*_Z_ denote the radial magnetic bearing force about the X*_f_*-axis and Z*_f_*-axis. *f*_Y_ is the axial magnetic bearing force. *α* and *β* are the rotation angle of the rotor about the X*_f_* -axis and Z*_f_* -axis, respectively. *α̇* and *β̇* are the angular velocity about the X*_f_* -axis and Z*_f_* -axis, respectively. Ω is the rotor speed relative to the rotor frame, and it is about Y*_f_* -axis. *i_AX_* and *i_AZ_* are the magnetic bearing control current about X*_f_* -axis and Z*_f_* -axis of the A-side.

Firstly, only one magnetically suspended SGCMG is analyzed. **M***^r^* is the torque acting on the rotor. By using the Euler equation, the magnetically suspended rotor dynamic model in the rotor frame can be obtained:

(1) $$M^{r}={\dot{H}}^{r}+\omega_{ir}^{r}\times H^{r}$$

In Equation (1), $\omega_{\mathrm{ir}}^{r}$ denotes the absolute angular velocity in rotor frame. It includes $\omega_{\mathrm{rf}}^{f}$, $\omega_{\mathrm{icmg}}^{\mathrm{cmg}}$, and **ω***^g^*. $\omega_{\mathrm{rf}}^{f}$ is the angular velocity of the rotor. It is the magnetic bearing frame relative to the rotor frame, and is shown in the magnetic bearing frame. $\omega_{\mathrm{icmg}}^{\mathrm{cmg}}$ is the angle velocity of CMG frame relative to the inertial frame. **ω***^g^* is the gimbal angular velocity:

(2) $$\omega_{\mathrm{ir}}^{r}=C_{f}^{r}\left(\omega_{\mathrm{rf}}^{f}+C_{g}^{f}C_{\mathrm{cmg}}^{g}\omega_{\mathrm{ig}}^{\mathrm{cmg}}\right)$$

The absolute angle velocity $\Omega_{\mathrm{ir}}^{r}$ of the rotor includes the spin rate **Ω***^r^* and the rate $\omega_{\mathrm{ir}}^{r}$ of the rotor frame:

(3) $$\Omega_{\mathrm{ir}}^{r}=\Omega^{r}+\omega_{\mathrm{ir}}^{r}=\Omega^{r}+C_{f}^{r}\left(\omega_{\mathrm{rf}}^{f}+C_{g}^{f}C_{\mathrm{cmg}}^{g}\omega_{\mathrm{ig}}^{\mathrm{cmg}}\right)$$

Because *α* and *β* are very small, then cos*α* ≈ 1, cos*β* ≈ 1, sin*α* ≈ *α*, sin*β* ≈ *β*, *αβ* ≈ 0. Then:

(4) $$\begin{array}{c} C_{f}^{r}=\left[\begin{array}{ccc} \text{cos}\beta & \text{sin}\beta & 0\\ -\text{sin}\beta & \text{cos}\beta & 0\\ 0 & 0 & 1 \end{array}\right]\left[\begin{array}{ccc} 1 & 0 & 0\\ 0 & \text{cos}\alpha & \text{sin}\alpha \\ 0 & -\text{sin}\alpha & \text{cos}\alpha \end{array}\right]\approx \left[\begin{array}{ccc} 1 & \beta & 0\\ -\beta & 1 & \alpha \\ 0 & -\alpha & 1 \end{array}\right],\omega_{rf}^{f}=\left[\begin{array}{c} \dot{\alpha }\\ 0\\ \dot{\beta } \end{array}\right]\\ C_{g}^{f}=\left[\begin{array}{ccc} \text{cos}45{}^{\circ} & 0 & -\text{sin}45{}^{\circ}\\ 0 & 1 & 0\\ \text{sin}45{}^{\circ} & 0 & \text{cos}45{}^{\circ} \end{array}\right] \end{array}$$

By rotating *δ* about X*_cmg_* axis from the CMG frame to the gimbal frame, then:

(5) $$C_{\mathrm{cmg}}^{g}=\left[\begin{array}{ccc} 1 & 0 & 0\\ 0 & \text{cos}\delta & \text{sin}\delta \\ 0 & -\text{sin}\delta & \text{cos}\delta \end{array}\right],\;\;\omega^{g}=\left[\begin{array}{c} \dot{\delta }\\ 0\\ 0 \end{array}\right],\;\;\;\;\;\;\;\omega_{\mathrm{ig}}^{\mathrm{cmg}}=\omega_{\mathrm{icmg}}^{\mathrm{cmg}}+\omega^{g}=\left[\begin{array}{c} \dot{\varphi }\\ \dot{\theta }\\ \dot{\phi } \end{array}\right]+\left[\begin{array}{c} \dot{\delta }\\ 0\\ 0 \end{array}\right]=\left[\begin{array}{c} \hat{\dot{\delta }}\\ \dot{\theta }\\ \dot{\phi } \end{array}\right]$$

where $\hat{\dot{\delta }}$, *θ̇* and *ϕ̇* show the absolute angular velocity of the CMG frame, and the detailed expression will be given in Section 2.2. By substituting the coordinate transformation matrix and the relative angular velocity, then:

(6) $$\Omega_{\mathrm{ir}}^{r}=\left[\begin{array}{c} \frac{\sqrt{2}}{2}\left(\hat{\dot{\delta }}-\dot{\phi }\text{cos}\delta +\dot{\theta }\text{sin}\delta \right)+\dot{\alpha }+\beta \left(\dot{\theta }\text{cos}\delta +\dot{\phi }\text{sin}\delta \right)\\ \Omega -\frac{\sqrt{2}}{2}\beta \left(\hat{\dot{\delta }}-\dot{\phi }\text{cos}\delta +\dot{\theta }\text{sin}\delta \right)-\dot{\alpha }\beta +\left(\dot{\theta }\text{cos}\delta +\dot{\phi }\text{sin}\delta \right)-\frac{\sqrt{2}}{2}\alpha \left(\hat{\dot{\delta }}+\dot{\phi }\text{cos}\delta -\dot{\theta }\text{sin}\delta \right)+\alpha \dot{\beta }\\ \frac{\sqrt{2}}{2}\left(\hat{\dot{\delta }}+\dot{\phi }\text{cos}\delta -\dot{\theta }\text{sin}\delta \right)+\dot{\beta }-\alpha \left(\dot{\theta }\text{cos}\delta +\dot{\phi }\text{sin}\delta \right) \end{array}\right]$$

In Equation (1), **H***^r^* is the rotor angular momentum in the rotor frame:

(7) $$H^{r}={\tilde{I}}_{r}\Omega_{\mathrm{ir}}^{r}=\left[\begin{array}{ccc} I_{\mathrm{rx}} & 0 & 0\\ 0 & I_{\mathrm{ry}} & 0\\ 0 & 0 & I_{\mathrm{rz}} \end{array}\right]\Omega_{\mathrm{ir}}^{r}$$

where **Ĩ***_r_* denotes the wheel moment of inertia in the spin direction. *I_rx_* and *I_ry_* are the radial inertia of the magnetically suspended rotor in the *x* and *y* direction, respectively. *I_rz_* is the axial inertia of the rotor. The angular momentum variation rate relative to the rotor frame can be obtained from the time derivatives:

(8) $${\dot{H}}^{r}=\left[\begin{array}{c} I_{rx}\left(\begin{array}{l} \frac{\sqrt{2}}{2}\left(\hat{\ddot{\delta }}-\ddot{\phi }\text{cos}\delta +\dot{\phi }\hat{\dot{\delta }}\text{sin}\delta +\ddot{\theta }\text{sin}\delta +\dot{\theta }\hat{\dot{\delta }}\text{cos}\delta \right)\\ +\dot{\beta }\dot{\theta }\text{cos}\delta +\beta \ddot{\theta }\text{cos}\delta -\beta \dot{\theta }\hat{\dot{\delta }}\text{sin}\delta +\dot{\beta }\dot{\phi }\text{sin}\delta +\beta \ddot{\phi }\text{sin}\delta +\beta \dot{\phi }\hat{\dot{\delta }}\text{cos}\delta +\ddot{\alpha } \end{array}\right)\\ I_{ry}\left(\begin{array}{l} \frac{\sqrt{2}}{2}\left(\begin{array}{l} -\dot{\beta }\hat{\dot{\delta }}-\beta \hat{\ddot{\delta }}+\dot{\beta }\dot{\phi }\text{cos}\delta +\beta \ddot{\phi }\text{cos}\delta -\beta \dot{\phi }\hat{\dot{\delta }}\text{sin}\delta -\dot{\beta }\dot{\theta }\text{sin}\delta -\beta \ddot{\theta }\text{sin}\delta -\beta \dot{\theta }\hat{\dot{\delta }}\text{cos}\delta \\ +\dot{\alpha }\hat{\dot{\delta }}+\alpha \hat{\ddot{\delta }}+\dot{\alpha }\dot{\phi }\text{cos}\delta +\alpha \ddot{\phi }\text{cos}\delta -\alpha \dot{\phi }\hat{\dot{\delta }}\text{sin}\delta -\dot{\alpha }\dot{\theta }\text{sin}\delta -\alpha \ddot{\theta }\text{sin}\delta -\alpha \dot{\theta }\hat{\dot{\delta }}\text{cos}\delta \end{array}\right)\\ +\dot{\Omega }+\ddot{\theta }\text{cos}\delta -\dot{\theta }\hat{\dot{\delta }}\text{sin}\delta +\ddot{\phi }\text{sin}\delta +\dot{\phi }\hat{\dot{\delta }}\text{cos}\delta -\ddot{\alpha }\beta +\alpha \ddot{\beta } \end{array}\right)\\ I_{rz}\left(\begin{array}{l} \frac{\sqrt{2}}{2}\left(\hat{\ddot{\delta }}+\ddot{\phi }\text{cos}\delta -\dot{\phi }\hat{\dot{\delta }}\text{sin}\delta -\ddot{\theta }\text{sin}\delta -\dot{\theta }\hat{\dot{\delta }}\text{cos}\delta \right)\\ -\dot{\alpha }\dot{\theta }\text{cos}\delta -\alpha \ddot{\theta }\text{cos}\delta +\alpha \dot{\theta }\hat{\dot{\delta }}\text{sin}\delta -\dot{\alpha }\dot{\phi }\text{sin}\delta -\alpha \ddot{\phi }\text{sin}\delta -\alpha \dot{\phi }\hat{\dot{\delta }}\text{cos}\delta +\ddot{\beta } \end{array}\right) \end{array}\right]$$

By substituting Equations (4)–(8) into Equation (1), the rotor torque in rotor frame can be obtained:

(9) $$M^{r}=\left[\begin{array}{c} -I_{\mathrm{ry}}\Omega \left(\frac{\sqrt{2}}{2}\left(\hat{\dot{\delta }}+\dot{\phi }\text{cos}\delta -\dot{\theta }\text{sin}\delta \right)+\dot{\beta }\right)+\left(I_{\text{rz}}-I_{\text{ry}}\right)\left(\dot{\theta }\text{cos}\delta +\dot{\phi }\text{sin}\delta \right)\left(\frac{\sqrt{2}}{2}\left(\hat{\dot{\delta }}+\dot{\phi }\text{cos}\delta -\dot{\theta }\text{sin}\delta \right)+\dot{\beta }\right)\\ +I_{\mathrm{rx}}\left(\frac{\sqrt{2}}{2}\left(\hat{\ddot{\delta }}-\ddot{\phi }\text{cos}\delta +\dot{\phi }\hat{\dot{\delta }}\text{sin}\delta +\ddot{\theta }\text{sin}\delta +\dot{\theta }\hat{\dot{\delta }}\text{cos}\delta \right)+\dot{\beta }\dot{\theta }\text{cos}\delta +\dot{\beta }\dot{\phi }\text{sin}\delta +\ddot{\alpha }\right)\\ \left(I_{\mathrm{rx}}-I_{\mathrm{rz}}\right)\left(\frac{\sqrt{2}}{2}\left(\hat{\dot{\delta }}+\dot{\phi }\text{cos}\delta -\dot{\theta }\text{sin}\delta \right)+\dot{\beta }\right)\left(\frac{\sqrt{2}}{2}\left(\hat{\dot{\delta }}-\dot{\phi }\text{cos}\delta +\dot{\theta }\text{sin}\delta \right)+\dot{\alpha }\right)\\ +I_{\mathrm{ry}}\left(\begin{array}{l} \frac{\sqrt{2}}{2}\left(-\dot{\beta }\hat{\dot{\delta }}+\dot{\beta }\dot{\phi }\text{cos}\delta -\dot{\beta }\dot{\theta }\text{sin}\delta +\dot{\alpha }\hat{\dot{\delta }}+\dot{\alpha }\dot{\phi }\text{cos}\delta -\dot{\alpha }\dot{\theta }\text{sin}\delta \right)\\ +\dot{\Omega }+\ddot{\theta }\text{cos}\delta -\dot{\theta }\hat{\dot{\delta }}\text{sin}\delta +\ddot{\phi }\text{sin}\delta +\dot{\phi }\hat{\dot{\delta }}\text{cos}\delta -\ddot{\alpha }\beta +\alpha \ddot{\beta } \end{array}\right)\\ I_{\mathrm{ry}}\Omega \left(\frac{\sqrt{2}}{2}\left(\hat{\dot{\delta }}-\dot{\phi }\text{cos}\delta +\dot{\theta }\text{sin}\delta \right)+\dot{\alpha }\right)+\left(I_{\text{ry}}-I_{\text{rx}}\right)\left(\dot{\theta }\text{cos}\delta +\dot{\phi }\text{sin}\delta \right)\left(\frac{\sqrt{2}}{2}\left(\hat{\dot{\delta }}-\dot{\phi }\text{cos}\delta +\dot{\theta }\text{sin}\delta \right)+\dot{\alpha }\right)\\ +I_{\mathrm{rz}}\left(\frac{\sqrt{2}}{2}\left(\hat{\ddot{\delta }}+\ddot{\phi }\text{cos}\delta -\dot{\phi }\hat{\dot{\delta }}\text{sin}\delta -\ddot{\theta }\text{sin}\delta -\dot{\theta }\hat{\dot{\delta }}\text{cos}\delta \right)-\dot{\alpha }\dot{\theta }\text{cos}\delta -\dot{\alpha }\dot{\phi }\text{sin}\delta +\ddot{\beta }\right) \end{array}\right]$$

From Equation (9), it can be seen that, for every magnetically suspended SGCMG, $I_{\text{ry}}\Omega \hat{\dot{\delta }}$ is the gyro coupled torque produced by the gimbal rotation relative to the inertial frame. $I_{\text{rr}}\hat{\ddot{\delta }}$ is the inertial coupled torque produced by the gimbal. *I*_ry_Ω*ϕ̇*cos*δ* and *I*_ry_Ω*θ̇*sin*δ* are the gyro coupled torque produced by the satellite rotation. *I*_rr_*ϕ̈*cos*δ* and *I*_rr_*θ̈*sin*δ* are the inertial coupled torque produced by the satellite rotation. *I*_ry_Ω*β̇* and *I*_ry_Ω*α̇* are the gyro coupled torque produced by the rotor tilt relative to the magnetic bearing. *I*_rr_*α̈* and *I*_rr_*β̈* are the inertial coupled torque produced by the rotor accelerating tilt relative to the magnetic bearing.

Equation (9) includes the gimbal angular velocity and the satellite angle acceleration. It shows that there is strong coupling between the rotor, the gimbal and the satellite. Moreover, there are many trigonometric functions, for example, sin*δ* and cos*δ*, which shows strongly nonlinear characteristics.

### 2.2. The Torque of Magnetically Suspended SGCMG Clusters

**Figure 4.**
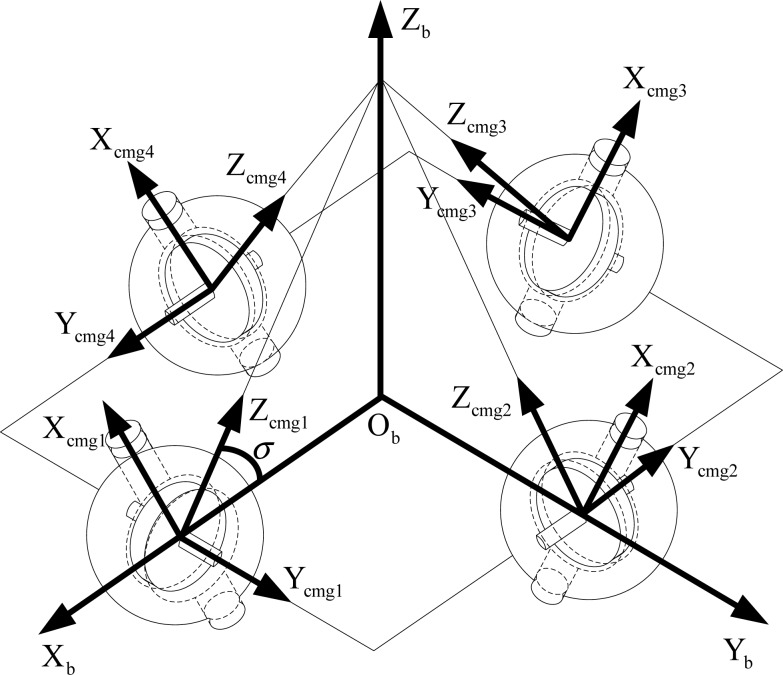
The pyramid configuration installation of four magnetically suspended SGCMGs.

In this paper, the pyramid configuration using four magnetically suspended SGCMGs is adopted (Figure 4). The frame of the *n*-th CMG can be obtained by rotating the satellite body frame *γ_n_* about the Z*_b_* axis, and then rotating *σ_n_* about the X*_b_* axis. **γ =** [*γ*_1_  *γ*_2_  *γ*_3_  *γ*_4_]^T^ = [90°  180°  270°  0]^T^, **σ** = [*σ*_1_  *σ*_2_  *σ*_3_  *σ*_4_]^T^ = [53.13°  53.13°  53.13°  53.13°  ]^T^.

By rotating the rotor frame 45° in the negative direction of the Y*_g_* axis, rotating −*δ* about the X*_g_* axis, and then transforming according to the installation matrix of the pyramid configuration, the attitude transformation matrix $C_{r}^{b}$ from rotor frame to satellite body frame can be obtained. Because the magnetic bearing gap is small, the rotation displacement can be ignored relative to the rotation of the gimbal and the satellite, then $C_{r}^{f}\approx E$:

(10) $$C_{r}^{b}=C_{\mathrm{cmg}}^{b}C_{g}^{\mathrm{cmg}}C_{f}^{g}C_{r}^{f}\approx C_{\mathrm{cmg}}^{b}C_{g}^{\mathrm{cmg}}C_{f}^{g}$$

where

(11) $$C_{\mathrm{cmg}}^{b}=\left[\begin{array}{ccc} \text{sin}\sigma \text{sin}\gamma & \text{cos}\gamma & -\text{cos}\sigma \text{sin}\gamma \\ -\text{sin}\sigma \text{cos}\gamma & \text{sin}\gamma & \text{cos}\sigma \text{cos}\gamma \\ \text{cos}\sigma & 0 & \text{sin}\sigma \end{array}\right],\;\;C_{g}^{\mathrm{cmg}}={\left(C_{\mathrm{cmg}}^{g}\right)}^{-1},\;\;C_{f}^{g}=\left[\begin{array}{ccc} \text{cos}45{}^{\circ} & 0 & \text{sin}45{}^{\circ}\\ 0 & 1 & 0\\ -\text{sin}45{}^{\circ} & 0 & \text{cos}45{}^{\circ} \end{array}\right]$$

The transformation matrix from gimbal frame to body frame is:

(12) $$C_{g}^{b}=C_{\mathrm{cmg}}^{b}C_{g}^{\mathrm{cmg}}=\left[\begin{array}{ccc} \text{sin}\sigma \text{sin}\gamma & \text{cos}\gamma \text{cos}\delta -\text{cos}\sigma \text{sin}\gamma \text{sin}\delta & -\text{cos}\gamma \text{sin}\delta -\text{cos}\sigma \text{sin}\gamma \text{cos}\delta \\ -\text{sin}\sigma \text{cos}\gamma & \text{sin}\gamma \text{cos}\delta +\text{cos}\sigma \text{cos}\gamma \text{sin}\delta & -\text{sin}\gamma \text{sin}\delta -\text{cos}\alpha \text{cos}\gamma \text{cos}\delta \\ \text{cos}\sigma & \text{sin}\sigma \text{sin}\delta & \text{sin}\sigma \text{cos}\delta \end{array}\right]$$

Equation (10) can be written as:

(13) $$C_{r}^{b}=\left[\begin{array}{ccc} \frac{\sqrt{2}}{2}\text{sin}\delta \text{cos}\gamma +\frac{\sqrt{2}}{2}\text{sin}\sigma \text{sin}\gamma +\frac{\sqrt{2}}{2}\text{cos}\delta \text{cos}\sigma \text{sin}\gamma & \text{cos}\delta \text{cos}\gamma -\text{sin}\delta \text{cos}\sigma \text{sin}\gamma & -\frac{\sqrt{2}}{2}\text{sin}\delta \text{cos}\gamma +\frac{\sqrt{2}}{2}\text{sin}\sigma \text{sin}\gamma -\frac{\sqrt{2}}{2}\text{cos}\delta \text{cos}\sigma \text{sin}\gamma \\ \frac{\sqrt{2}}{2}\text{sin}\delta \text{sin}\gamma -\frac{\sqrt{2}}{2}\text{sin}\sigma \text{cos}\gamma -\frac{\sqrt{2}}{2}\text{cos}\delta \text{cos}\sigma \text{cos}\gamma & \text{cos}\delta \text{sin}\gamma +\text{sin}\delta \text{cos}\sigma \text{cos}\gamma & -\frac{\sqrt{2}}{2}\text{sin}\delta \text{sin}\gamma -\frac{\sqrt{2}}{2}\text{sin}\sigma \text{cos}\gamma +\frac{\sqrt{2}}{2}\text{cos}\delta \text{cos}\sigma \text{cos}\gamma \\ \frac{\sqrt{2}}{2}\text{cos}\sigma -\frac{\sqrt{2}}{2}\text{cos}\delta \text{sin}\sigma & \text{sin}\delta \text{sin}\sigma & \frac{\sqrt{2}}{2}\text{cos}\sigma +\frac{\sqrt{2}}{2}\text{cos}\delta \text{sin}\sigma \end{array}\right]$$

Then, the absolute angular velocity of the CMG frame can be obtained:

(14) $$C_{b}^{\mathrm{cmg}}\omega_{\mathrm{ib}}^{b}+\omega^{g}=\left[\begin{array}{ccc} \text{sin}\sigma \text{sin}\gamma & -\text{sin}\sigma \text{cos}\gamma & \text{cos}\sigma \\ \text{cos}\gamma & \text{sin}\gamma & 0\\ -\text{cos}\sigma \text{sin}\gamma & \text{cos}\sigma \text{cos}\gamma & \text{sin}\sigma \end{array}\right]\left[\begin{array}{c} \omega_{\mathrm{ibx}}^{b}\\ \omega_{\mathrm{iby}}^{b}\\ \omega_{\mathrm{ibz}}^{b} \end{array}\right]+\left[\begin{array}{c} \dot{\delta }\\ 0\\ 0 \end{array}\right]$$

The torque generated by the rotor in body frame can be obtained after the sum of four CMGs:

(15) $$M^{b}=\sum_{n=1}^{4}C_{r,n}^{b}M_{n}^{r}$$

By Equation (15), **M***^b^*, the torque of the magnetically suspended SGCMG cluster acting on the three inertial principal axis of the satellite can be obtained.

### 2.3. Satellite Attitude Dynamic Model Including Magnetically Suspended SGCMGs

The satellite attitude dynamic equation including magnetically suspended SGCMGs can be obtained by the law of angular momentum conservation:

(16) $$J{\dot{\omega }}_{\mathrm{ib}}^{b}+\omega_{\mathrm{ib}}^{b}\times \left(J\omega_{\mathrm{ib}}^{b}\right)+M^{b}=u_{d}$$

where **J** is moment of inertia of the satellite. $\omega_{\mathrm{ib}}^{b}$ is the angular velocity vector relative to the inertia frame, and it is shown in the body frame. **u***_d_* is the environmental disturbance torque, such as aerodynamic drag gravity gradient, solar radiation pressure and Earth magnetic torque. $\omega_{\mathrm{ib}}^{b}\times \left(J\omega_{\mathrm{ib}}^{b}\right)$ represents the gyroscope torque generated by the satellite rotation.

Equation (16) can be simplified to $J{\dot{\omega }}_{\mathrm{ib}}^{b}=u_{c}+u_{d}$, where **u***_c_* is the command control torque acting on the satellite. The command torque for magnetically suspended SGCMGs is:

(17) $$u_{g}=-u_{c}-\omega_{\mathrm{ib}}^{b}\times H_{g}-\omega_{\mathrm{ib}}^{b}\times \left(J\omega_{\mathrm{ib}}^{b}\right)$$

where **H***_g_* is the total angular momentum of the actuator relative to the satellite body frame. The gimbal angle of four magnetically suspended SGCMGs is **δ** = [*δ*_1_  *δ*_2_  *δ*_3_  *δ*_4_]^T^, where *δ_n_* represents the gimbal angle of the *n* -th CMG. By substituting **γ** into Equation (12), the total momentum of four magnetically suspended SGCMGs is:

(18) $$H_{g}=h_{0}\left[\begin{array}{c} -\text{cos}\sigma_{1}\text{sin}\delta_{1}-\text{cos}\delta_{2}+\text{cos}\sigma_{3}\text{sin}\delta_{3}+\text{cos}\delta_{4}\\ \text{cos}\delta_{1}-\text{cos}\sigma_{2}\text{sin}\delta_{2}-\text{cos}\delta_{3}+\text{cos}\sigma_{4}\text{sin}\delta_{4}\\ \text{sin}\sigma_{1}\text{sin}\delta_{1}+\text{sin}\sigma_{2}\text{sin}\delta_{2}+\text{sin}\sigma_{3}\text{sin}\delta_{3}+\text{sin}\sigma_{4}\text{sin}\delta_{4} \end{array}\right]$$

where *h*_0_ = *I_ry_*Ω denotes the rotor angular momentum. Then the variation rate of CMG angular momentum in the satellite body frame can be obtained:

(19) $$u_{g}={\dot{H}}_{g}=C\left(\delta \right)\dot{\delta }$$

where **C**(**δ**) is:

(20) $$C\left(\delta \right)=\left[\begin{array}{cccc} -\text{cos}\sigma_{1}\text{cos}\delta_{1} & \text{sin}\delta_{2} & \text{cos}\sigma_{3}\text{cos}\delta_{3} & -\text{sin}\delta_{4}\\ -\text{sin}\delta_{1} & -\text{cos}\sigma_{2}\text{cos}\delta_{2} & \text{sin}\delta_{3} & \text{cos}\sigma_{4}\text{cos}\delta_{4}\\ \text{sin}\sigma_{1}\text{cos}\delta_{1} & \text{sin}\sigma_{2}\text{cos}\delta_{2} & \text{sin}\sigma_{3}\text{cos}\delta_{3} & \text{sin}\sigma_{4}\text{cos}\delta_{4} \end{array}\right]$$

The command gimbal rate is calculated by the robust pseudo-inverse steering law with null motion [15]:

(21) $$\dot{\delta }={\dot{\delta }}_{C}+{\dot{\delta }}_{N}$$

where **δ̇***_C_* denotes the command value calculated by robust pseudo-inverse steering law, **δ̇***_N_* denotes the command value calculated by null motion.

The attitude kinematics are described with the quaternion of the satellite body frame relative to the orbit frame, and then the attitude kinematics equation is:

(22) $$\dot{q}=\frac{1}{2}q⊗\omega_{\mathrm{ob}}$$

where **q** = [*q*_0_  *q*_1_  *q*_2_  *q*_3_]*^T^* represents the attitude quaternion, ⊗ means quaternion multiply. The satellite body rate relative to the orbit is **ω***_ob_* = **ω***_b_* − *A***ω***_o_*. *A* is the attitude rotation matrix relative to the orbit frame. **ω***_o_* = [0  −*ω*_0_  0]*^T^* is the orbit velocity.

## 3. Feedforward Compensation for the Rotor Disturbance Torque

**Figure 5.**
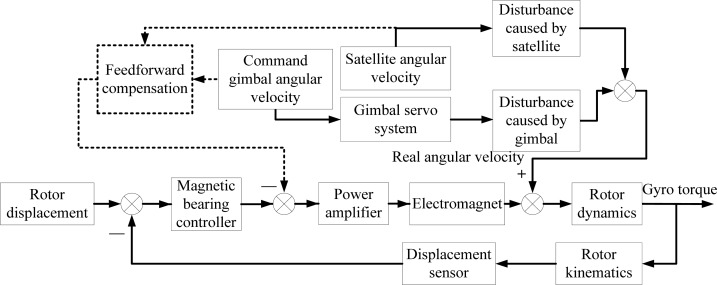
Sketch of the feedforward compensation.

When the satellite maneuvers quickly, the satellite angular velocity and gimbal rate will introduce extra torque on the rotor, and the rotor tilt motion will be enhanced. The disturbance torque can be compensated by the feedforward control. Figure 5 gives a diagram of the feedforward compensation.

In order to keep the rotor displacement close to zero, the controller provides the control voltage to maintain the rotor stability. However, the rotation of satellite and gimbal introduces disturbances to the rotor. Feedforward compensation is used to reduce the influence on the rotor. The compensation does not change the control system stability.

The disturbance caused by satellite and gimbal is given in Equation (9). When the satellite body and the gimbal are fixed, *δ̇*, *ϕ̇* and *θ̇* are all zero. The torque is generated by the rotor. Equation (9) is written as:

(23) $$M^{r}\left(0\right)=\left[\begin{array}{c} -I_{\mathrm{ry}}\Omega \dot{\beta }+I_{\mathrm{rx}}\ddot{\alpha }\\ \left(I_{\mathrm{rx}}-I_{\mathrm{rz}}\right)\dot{\alpha }\dot{\beta }+I_{\text{ry}}\left(\dot{\Omega }-\ddot{\alpha }\beta +\alpha \ddot{\beta }\right)\\ I_{\mathrm{ry}}\Omega \dot{\alpha }+I_{\mathrm{rz}}\ddot{\beta } \end{array}\right]$$

Assuming that the rotor acceleration is zero, Ω̇ = 0, the rotational inertia of the non-rotation axis is uniform, *I_G_* = *I_O_* = *I_rr_*, *I_S_* = *I_rz_*. By ignoring the first order item that includes *α* and *β*, the above equation can be simplified as:

(24) $$M^{r}\left(0\right)=\left[\begin{array}{c} -I_{\mathrm{ry}}\Omega \dot{\beta }+I_{\mathrm{rr}}\ddot{\alpha }\\ 0\\ I_{\mathrm{ry}}\Omega \dot{\alpha }+I_{\mathrm{rr}}\ddot{\beta } \end{array}\right]$$

By deleting **M***^r^* (0) from **M***^r^*, the equivalent disturbance torque $M_{\mathrm{ext}}^{r}$ acting on the magnetic bearing control system can be obtained:

(25) $$M_{\mathrm{ext}}^{r}=\left[\begin{array}{c} -\frac{\sqrt{2}}{2}I_{\mathrm{ry}}\Omega \left(\hat{\dot{\delta }}+\dot{\phi }\text{cos}\delta -\dot{\theta }\text{sin}\delta \right)+\frac{\sqrt{2}}{2}I_{\mathrm{rr}}\left(\hat{\ddot{\delta }}-\ddot{\phi }\text{cos}\delta +\dot{\phi }\hat{\dot{\delta }}\text{sin}\delta +\ddot{\theta }\text{sin}\delta +\dot{\theta }\hat{\dot{\delta }}\text{cos}\delta \right)\\ 0\\ \frac{\sqrt{2}}{2}I_{\mathrm{ry}}\Omega \left(\hat{\dot{\delta }}-\dot{\phi }\text{cos}\delta +\dot{\theta }\text{sin}\delta \right)+\frac{\sqrt{2}}{2}I_{\mathrm{rr}}\left(\hat{\ddot{\delta }}+\ddot{\phi }\text{cos}\delta -\dot{\phi }\hat{\dot{\delta }}\text{sin}\delta -\ddot{\theta }\text{sin}\delta -\dot{\theta }\hat{\dot{\delta }}\text{cos}\delta \right) \end{array}\right]$$

Since *I_rz_*Ω >> *I_rr_*, the gimbal and satellite have more influence on the rotor than their angle acceleration. It is to say, the gyro coupling torque is more obvious than the inertia coupling torque. In designing the steering law, the limited gimbal angular velocity must be considered to prevent the instability. The gimbal angle acceleration should be limited to improve the dynamic response ability.

## 4. Simulation Results and Analysis

The initial satellite attitude angle is [−20°  50°  30°]*^T^*. The target satellite attitude angle is [0°  0°  0°]*^T^*. The moment of inertia of the satellite is [12  12  6]^T^ kg · m^2^. The initial gimbal angle is [90°  −90°  90°  −90°]*^T^*. The radial inertia of the magnetically suspended rotor is *I_rx_* = *I_ry_* = *I_rr_* = 0.0034kg · m^2^, the axial inertia is *I_rz_* = 0.0052kg · m^2^, the speed of the rotor is 15,000 r/min. Ω = 500*π*rad/s.

### 4.1. The Comparison of with and without Feedforward Compensation not Using an Attitude Control Loop

**Figure 6.**
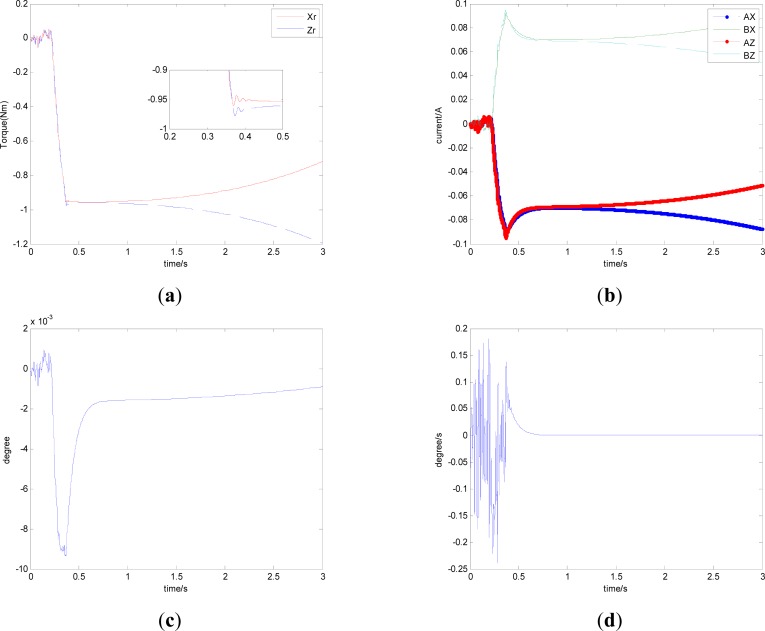

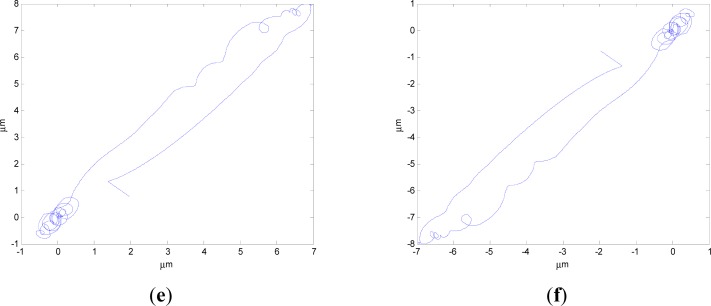
The curves without feedforward compensation. (**a**) Magnetic bearing torque; (**b**) Control current; (**c**) The rotor tilt angle; (**d**) The rotor tilt angle velocity; (**e**) The rotor displacement of A-side; (**f**) The rotor displacement of B-side.

**Figure 7.**
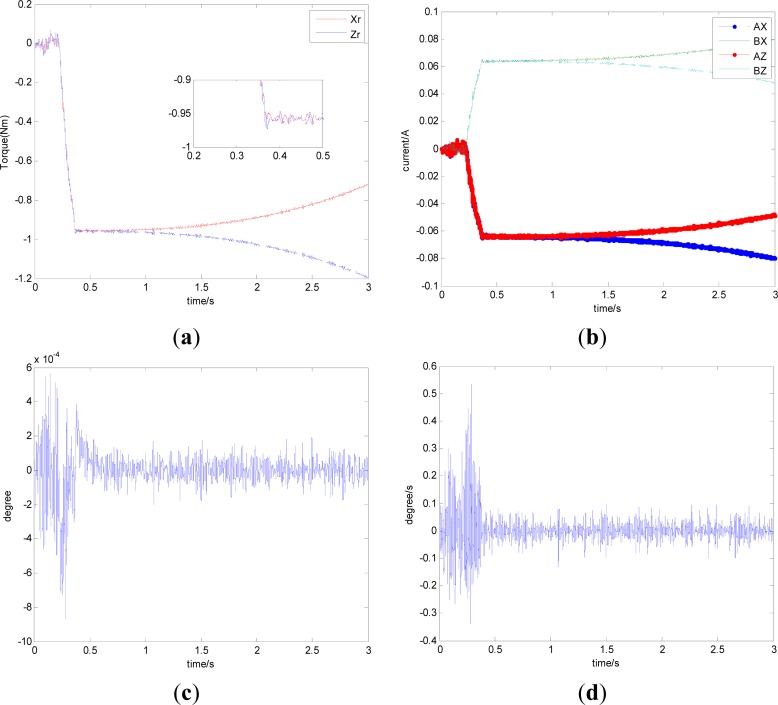

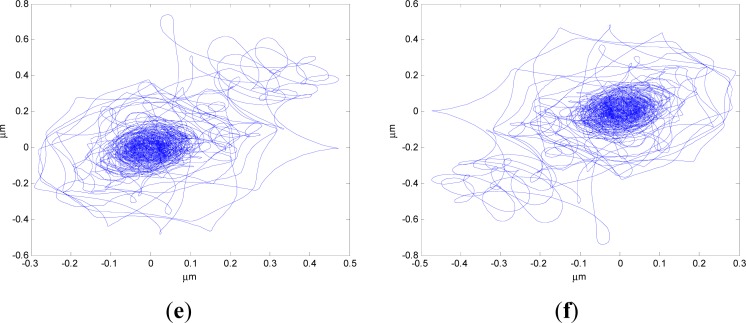
The curves with feedforward compensation. (**a**) Magnetic bearing torque; (**b**) Control current; (**c**) The rotor tilt angle (**d**) The rotor tilt angle velocity; (**e**) The rotor displacement of A-side (**f**) The rotor displacement of B-side; (**e**) The rotor displacement of A-side; (**f**) The rotor displacement of B-side.

In this section, aiming at the dynamic characteristics of a single gimbal magnetically suspended CMG, the simulation is performed without the attitude control loop. The gimbal acceleration is 120°/s^2^, the maximal gimbal rate is 10°/s, and the simulation period is 3 s. The gimbal rate limit is *δ̇*_max_ = 10°/s. Figures 6 and 7 are the simulation results of with and without feedforward compensation. From the local zoom in of Figures 6(a) and 7(a), it can been seen that, the vibration displacement range is decreased to some degree after using the feedforward compensation, and then a faster response and the higher precision of the output torque can be obtained. From the comparison of Figure 6(b) with Figure 7(b), it can be seen that the overshooting of control current is reduced by using feedforward compensation, and the power consumption is decreased. Because the magnetic bearing control frequency is highly relative to the satellite motion, the disturbance torque induced from the satellite angle velocity can be compensated by the feedforward loop.

Figures 6(c) and 7(c) give the extent of tilt of the rotor relative to the magnetic bearing. It can be seen that after using feedforward compensation, the tilt extent is reduced, but the relative tilt angle velocity is increased, which can be seen from Figures 6(d) and 7(d). Figures 6(e,f) and 7(e,f) correspond to the displacement of the two sides of the rotor. It can be seen that the rotor displacement in the maneuvering process is reduced apparently by the feedforward compensation.

### 4.2. The Comparison of with and without Feedforward Compensation Using Attitude Control Loop

**Figure 8.**
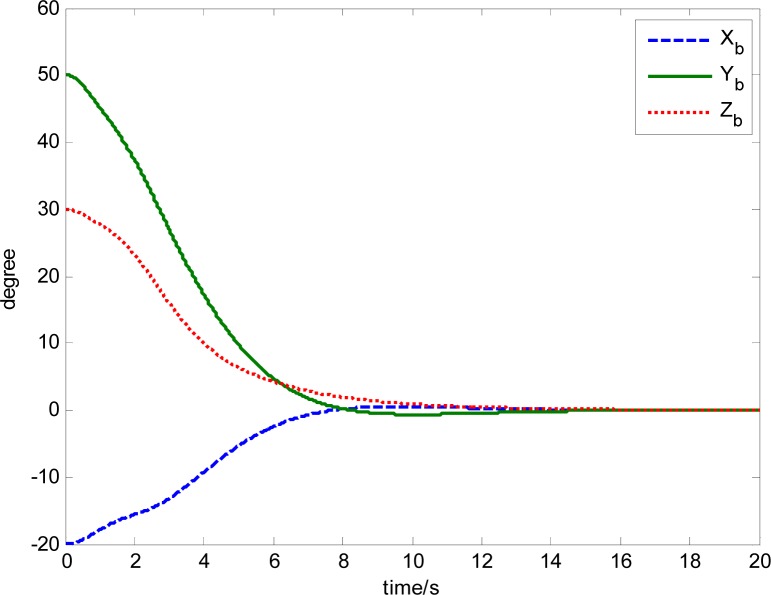
Satellite attitude angle.

**Figure 9.**
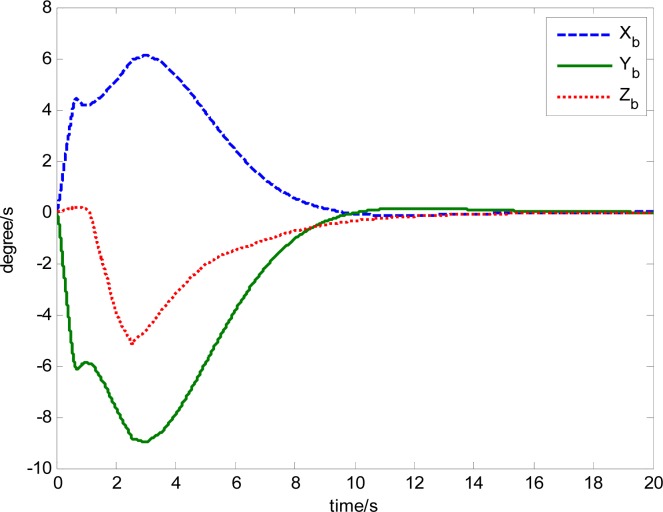
Satellite angle velocity.

**Figure 10.**
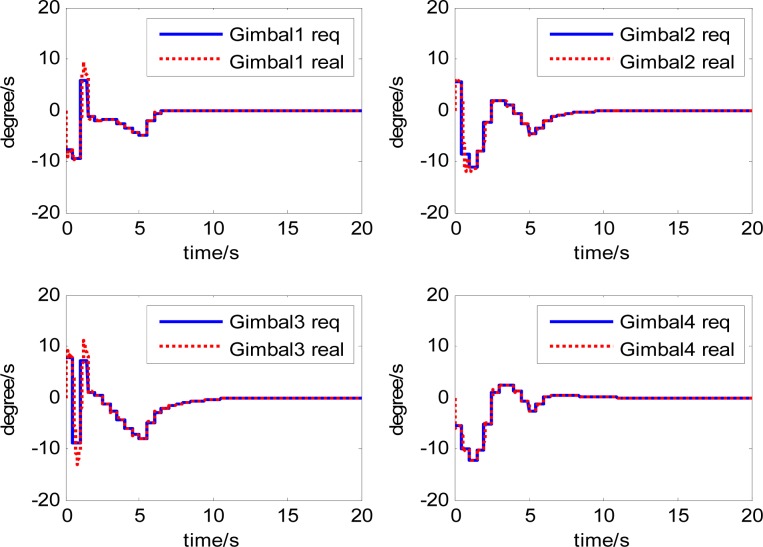
The gimbal rates of magnetically suspended SGCMGs.

**Figure 11.**
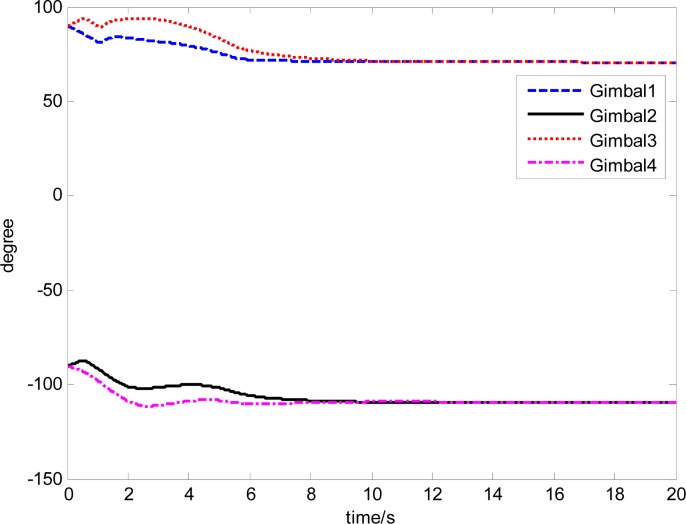
The gimbal angle of magnetically suspended SGCMGs.

**Figure 12.**
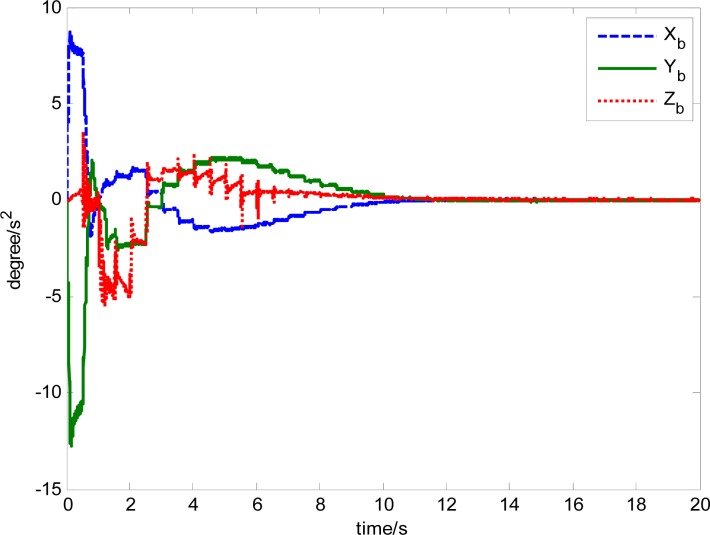
Satellite angle acceleration.

**Figure 13.**
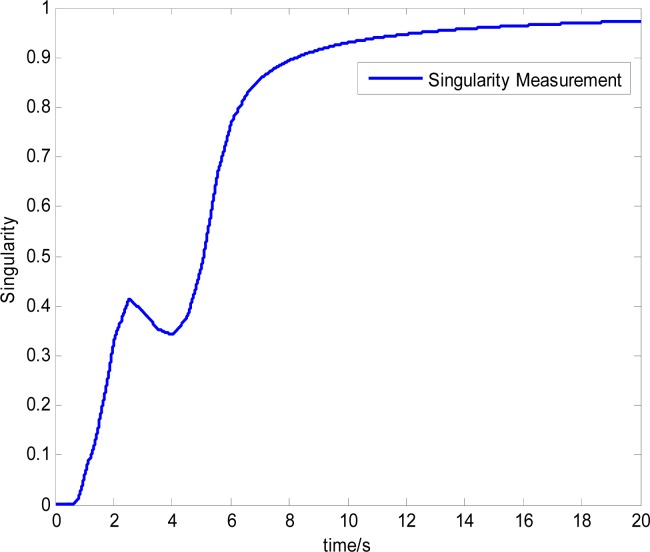
Singularity measurement curves.

The attitude control period is 0.5 s. The maximal gimbal rate is *δ̇*_max_ = 10°/s. The simulation results of the three-axis attitude control of the satellite with feedforward compensation are given in Figures 8–13. Figure 8 is the satellite attitude angle. Figure 9 is the satellite angle velocity. Figure 10 gives the gimbal rates of four magnetically suspended SGCMGs. Figure 11 gives the gimbal angles of four magnetically suspended SGCMGs. Figure 12 is the satellite angle acceleration.

The magnetic bearing force in each channel depends on the magnetic bearing coefficient, the magnetic gap of the two sides of the magnetic bearing, the bias current, the rotor displacement and the control current, *etc*. When the magnetic bearing design is finished, it is determined by the magnetic bearing coefficient and magnetic bearing gap. By adjusting the control current, the magnetic bearing force is changed to assure the convergence of rotor displacement, but the magnetic bearing force has a nonlinear relationship with the rotor displacement and the control current. The magnetic bearing controller is used to make sure the stability under the working point.

**Figure 14.**
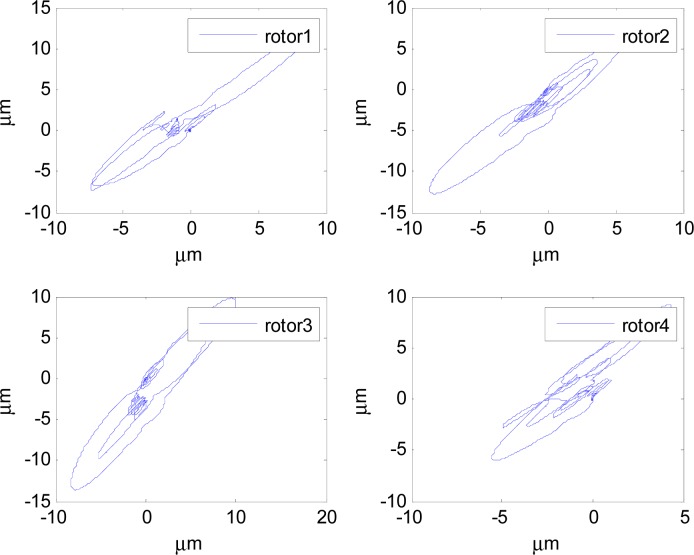
The rotor displacement without feedforward compensation.

**Figure 15.**
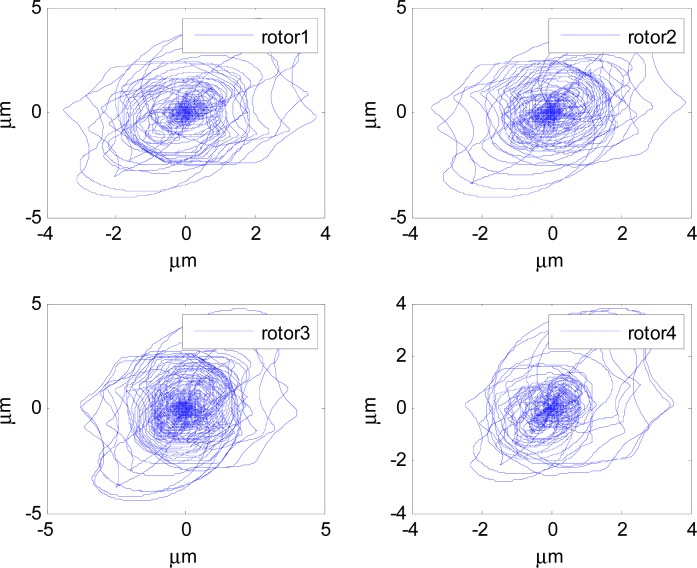
The rotor displacement with feedforward compensation.

**Figure 16.**
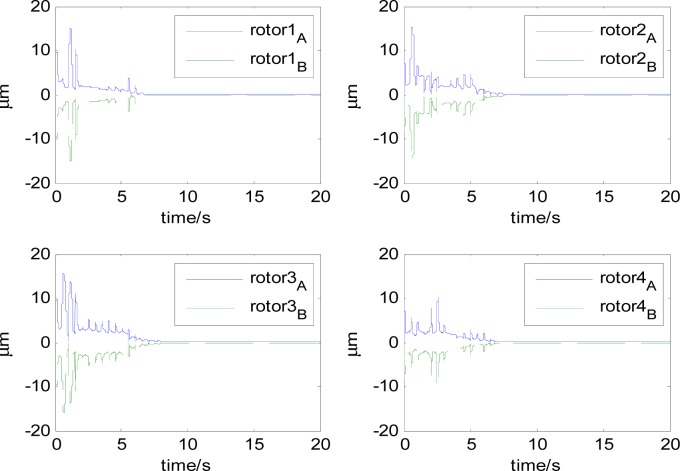
The magnitude of the rotor displacement without feedforward compensation.

**Figure 17.**
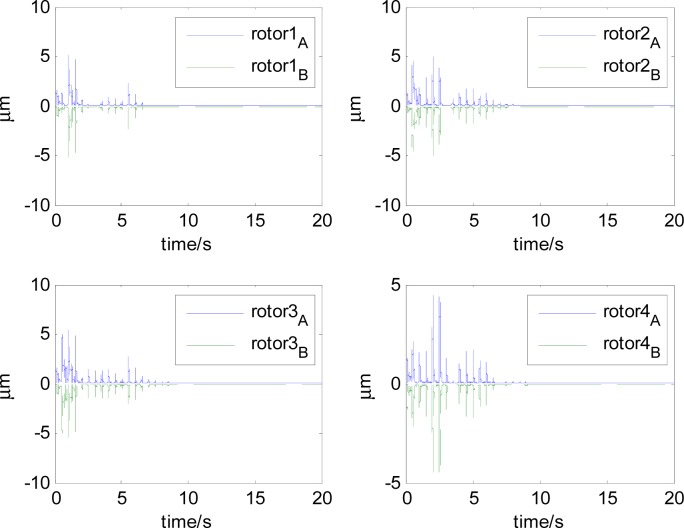
The magnitude of the rotor displacement with feedforward compensation.

**Figure 18.**
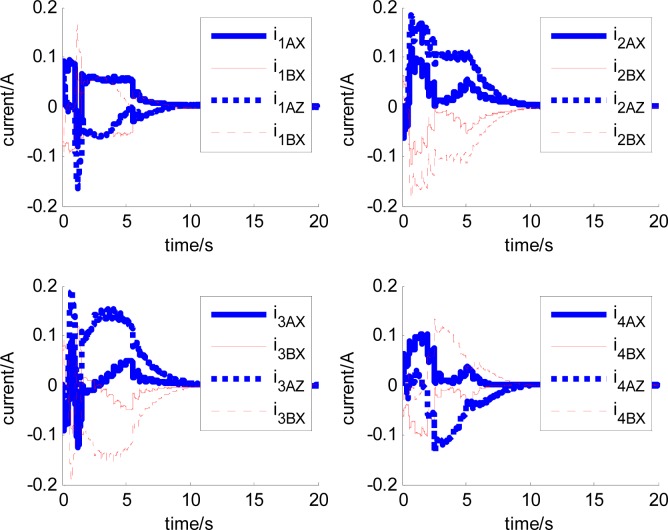
The magnetically suspended rotor control current without feedforward compensation.

**Figure 19.**
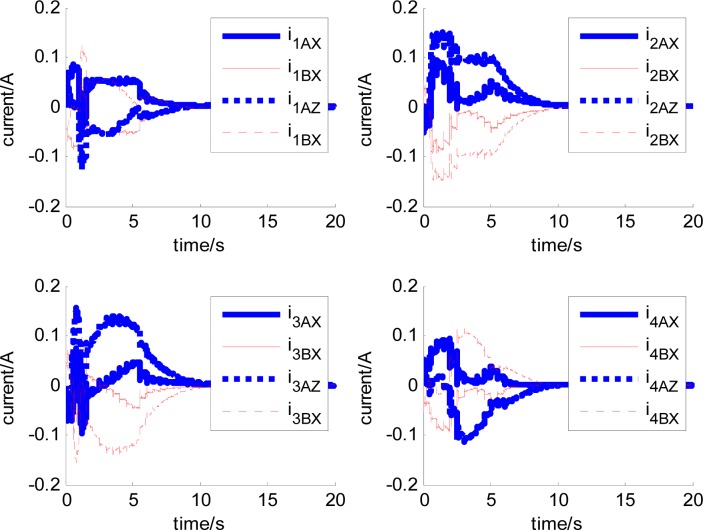
The magnetically suspended rotor control current with feedforward compensation.

In Figures 14–19, the left column gives the rotor X–Y displacement, the magnitude of rotor displacement and control current without feedforward compensation. The right column gives the results with feedforward compensation.

**Figure 20.**
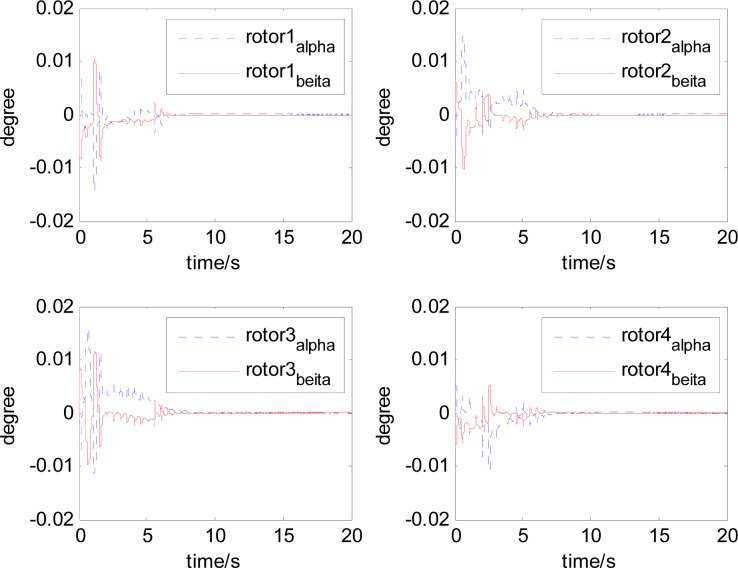
The magnetically suspended rotor tilt curves relative to rotor center before compensation.

**Figure 21.**
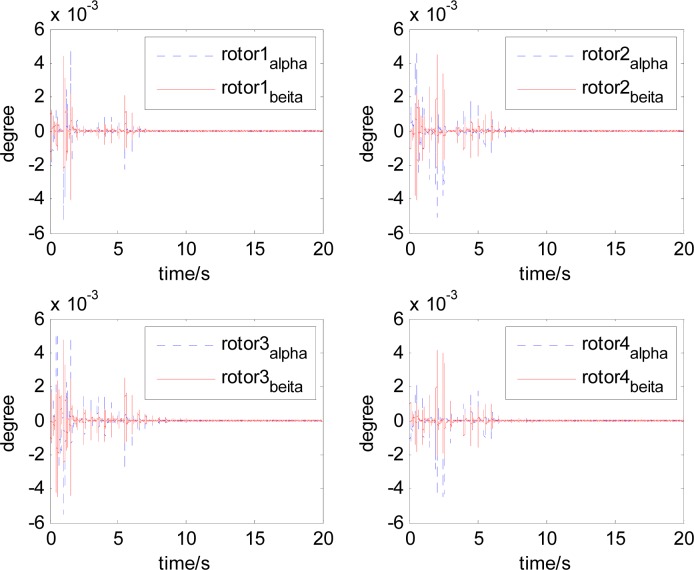
The magnetically suspended rotor tilt curves relative to rotor center after compensation.

**Figure 22.**
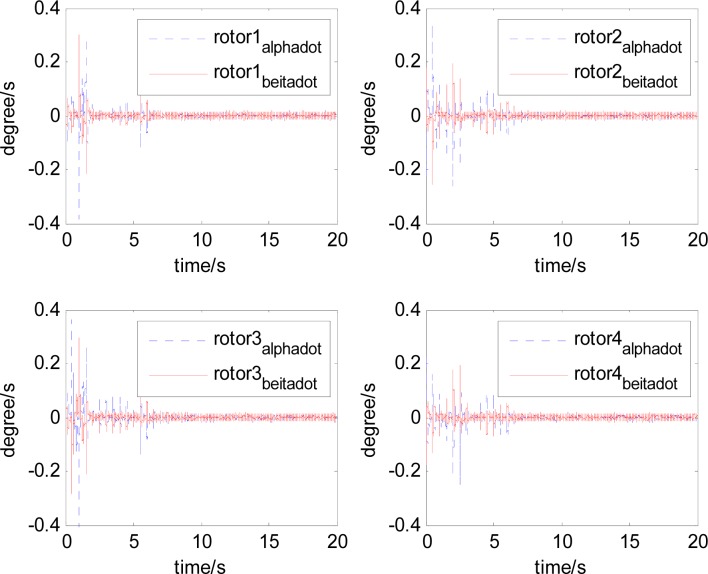
The magnetically suspended rotor angle velocity relative to rotor center before compensation.

**Figure 23.**
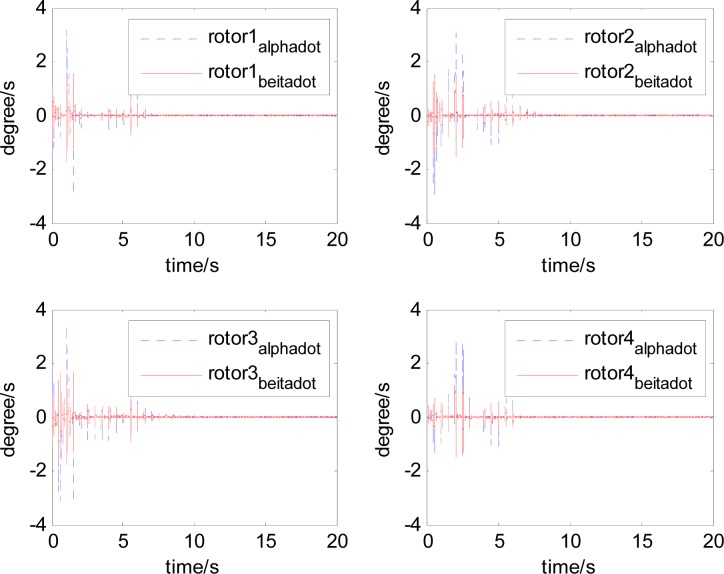
The magnetically suspended rotor angle velocity relative to rotor center after compensation.

It can be seen that, after using feedforward compensation, the rotor displacement is reduced to about 30%, which decreases the risk of the rotor instability. Figures 18–19 show that the control current does not vary apparently. Although the rotor displacement and the given control current are reduced, extra control current is needed for feedforward compensation. It is to say, the command torque is invariant. The torque is output by the control current to assure the stable suspending of the rotor, and so the control current did not vary apparently. By using feedforward compensation, the maximal displacement of the rotor is less than 10% of the magnetic bearing protecting gap, which can ensure the requirement of the stability of magnetically suspended rotor. Figures 20–23 give *α*, *β*, *α̇* and *β̇* of the magnetically suspended SGCMG rotor, which describe the motion of the rotor relative to the magnetic bearing.

**Figure 24.**
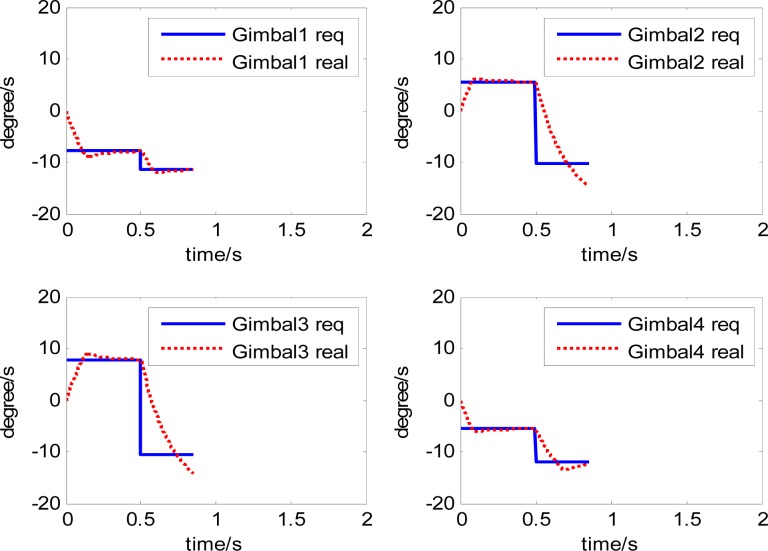
The gimbal rate of four magnetically suspended SGCMGs.

**Figure 25.**
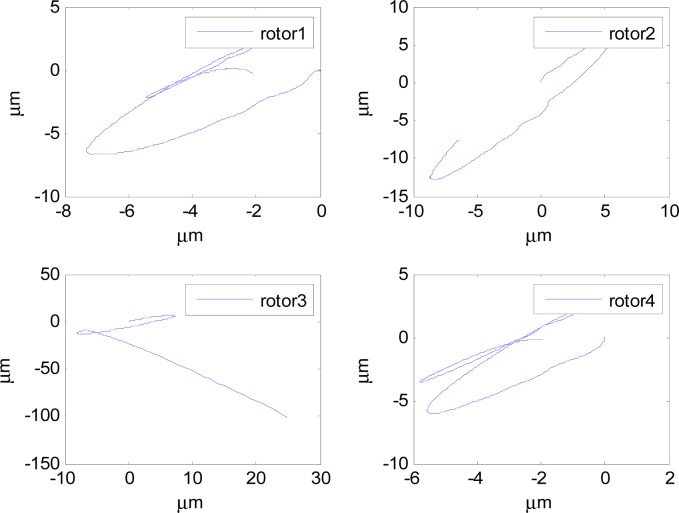
The rotor X–Y displacement without feedforward compensation.

By using feedforward compensation for the magnetically suspended control system, the response of angle acceleration and angle velocity becomes faster owing to the increase of the control current, which decreases the rotor angle displacement. There is a magnetic saturation problem in the magnetic bearing design, so there exists a magnetic bearing control current saturation limit. When the satellite and the gimbal rate exceed the limit, the additional disturbance torque on the rotor is large, which exceeds the extent of the control current, and then the rotor becomes instable, even resulting in CMG failure. This will influence the stability of the whole system. When the maximal gimbal rate limit is *δ̇*_max_ = 12°/s, the results of not using feedforward compensation are shown in Figures 24 and 25.

The terminative condition of the simulation is whether the rotor displacement exceeds the magnetic gap (100 μm) or not. The above figures show that, the simulation finish in less than 1 s. The reason is that magnetically suspended SGCMG3 becomes instable. Because the gimbal angular acceleration command limit is not performed, the overshooting of gimbal servo system results in the real gimbal rate being larger than 12°/s, and then the equivalent additive magnetic bearing torque is large, which results in an increase of the rotor displacement. There exists the magnetic bearing control current saturation limit, it is difficult to provide sufficient magnetic bearing torque, which results in the rotor displacement exceeding the approximately linear zone and reaching the magnetic gap instantaneously, and then the magnetically suspended rotor becomes instable. In the end, the stability of magnetic bearing control system and satellite control system are destroyed.

**Figure 26.**
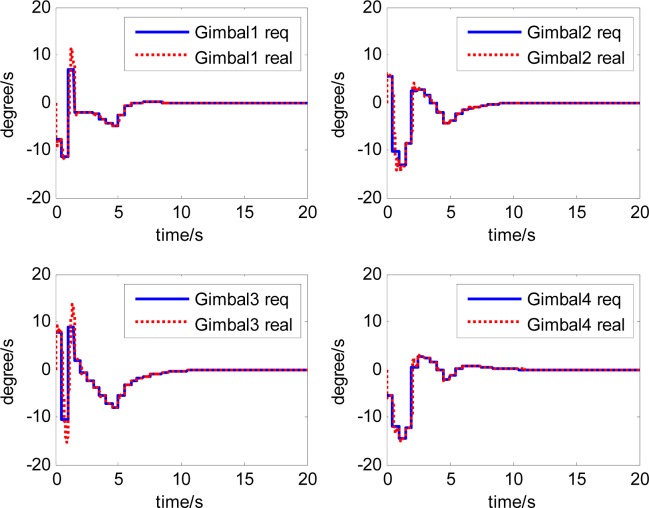
The gimbal rate of four magnetically suspended SGCMGs with feedforward compensation.

**Figure 27.**
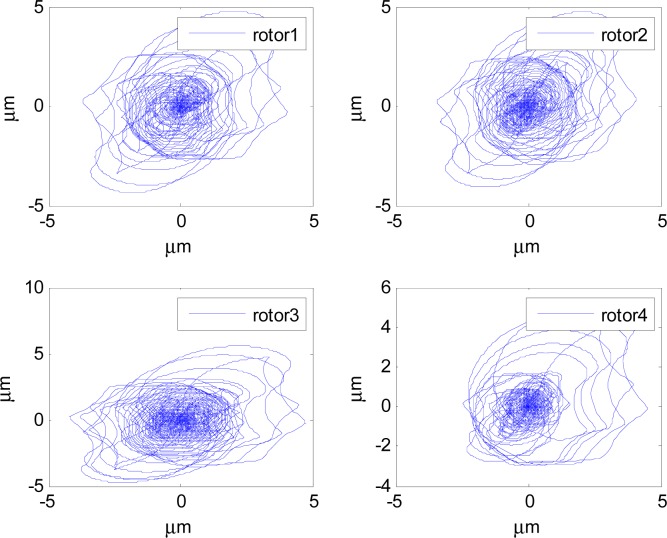
The rotor displacement with feedforward compensation.

The curves of gimbal rate and rotor displacement with feedforward compensation are given in Figures 26 and 27.

It can be seen that the rotor displacement is reduced by the feedforward compensation. Although there is overshooting of the gimbal rate, even exceeding 14°/s, the displacement of the rotor is less than 5 μm. This ensures the normal use of the magnetically suspended SGCMG. There are also some research results that are related to the compensation and disturbance attenuation [16–19]. In the future, the advanced algorithm will be employed.

### 4.3. Analysis on the Output Torque

**Figure 28.**
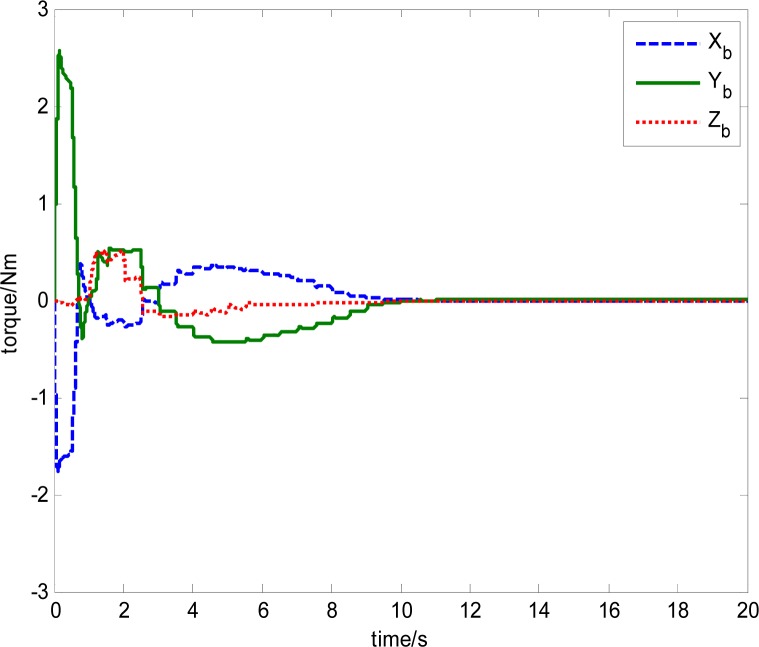
Torque calculated using Equation (19).

**Figure 29.**
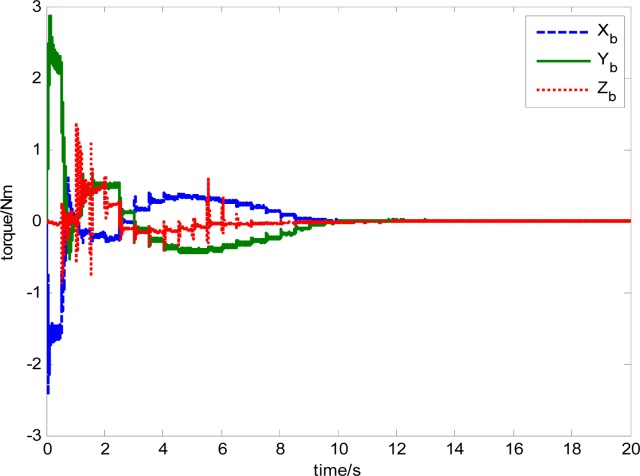
Torque calculated using Equation (15).

The output torque of magnetically suspended SGCMG includes the inertial coupled torque and the gyro coupled torque, which is produced from the coupling of the satellite, the gimbal and the rotor. Traditionally, the inertial coupled torque is ignored when Equation (19) is used to calculate the output torque according to the Jacobian matrix and the gimbal rate. In this paper, the total torque is produced from the magnetically suspended rotor dynamic equation, as shown in Equation (15). By using this method, a more actual output torque of the actuator can be obtained. This is important for the analysis of the agile satellite attitude control system. Equation (15) also includes the strong coupling relationship between the rotor, gimbal, and satellite. This results in strongly nonlinear in the trigonometric function item that is relative with the gimbal angle. Figure 28 shows the torque calculated from Equation (19). Figure 29 gives the total output torque calculated from Equation (15).

From the simulation results, it can be seen that the trend of torque curves calculated using Equations (15) and (19) are consistent. Figure 29 shows the influence of the rotor imbalance vibration and gimbal system, which is high frequency compared with the satellite attitude control system, so in the simulation, the torque model in Equation (15) should be used, which can emulate the dynamic characteristics of a magnetically suspended SGCMG more really. In the feedforward compensation for a magnetically suspended rotor, the compensation should be performed according to the rotation rate of the CMG frame with respect to the inertial frame, so in a situation of satellite rapid maneuver, the dynamic modeling of the magnetically suspended SGCMG is very important. The simulation results show that it coincides with the theoretical analysis results.

## 5. Conclusions

With magnetically suspended SGCMGs mounted as a pyramid, the modeling of satellite attitude dynamic is built, and feedforward compensation control is used. The simulation results are given to show that by using the feedforward control, the displacement of the rotor is reduced. This is important for the future semi-physics experiment, and becomes the basis for the application of a single gimbal magnetically suspended CMG in agile satellites.
